# Dendritic Cell-Directed Vaccination with a Lentivector Encoding PSCA for Prostate Cancer in Mice

**DOI:** 10.1371/journal.pone.0048866

**Published:** 2012-11-06

**Authors:** Liang Xiao, Kye-Il Joo, Matthew Lim, Pin Wang

**Affiliations:** 1 Mork Family Department of Chemical Engineering and Materials Science, University of Southern California, Los Angeles, California, United States of America; 2 Department of Biomedical Engineering, University of Southern California, Los Angeles, California, United States of America; 3 Department of Pharmacology and Pharmaceutical Sciences, University of Southern California, Los Angeles, California, United States of America; Istituto Superiore di Sanità, Italy

## Abstract

Many studies have demonstrated that prostate stem cell antigen (PSCA) is an attractive target for immunotherapy based on its overexpression in prostate tumor tissue, especially in some metastatic tissues. In this study, we evaluated dendritic cell (DC)-directed lentiviral vector (DCLV) encoding murine PSCA (DCLV-PSCA) as a novel tumor vaccine for prostate cancer in mouse models. We showed that DCLV-PSCA could preferentially deliver the PSCA antigen gene to DC-SIGN-expressing 293T cells and bone marrow-derived DCs (BMDCs). Direct immunization with the DCLV-PSCA in male C57BL/6 mice elicited robust PSCA-responsive CD8^+^ and CD4^+^ T cells *in vivo*. In a transgenic adenocarcinoma mouse prostate cell line (TRAMP-C1) synergetic tumor model, we further demonstrated that DCLV-PSCA-vaccinated mice could be protected from lethal tumor challenge in a prophylactic model, whereas slower tumor growth was observed in a therapeutic model. This DCLV-PSCA vaccine also showed efficacy in inhibiting tumor metastases using a PSCA-expressing B16-F10 model. Taken together, these data suggest that DCLV is a potent vaccine carrier for PSCA in delivering anti-prostate cancer immunity.

## Introduction

In 2011, the FDA approved the first therapeutic cancer vaccine for treatment of asymptomatic or slightly symptomatic hormone refractory prostate cancer [1], [2], a great encouragement for both prostate cancer patients and scientists working on cancer immunotherapy. Immunologic therapies can instruct the immune system to recognize and eliminate tumor cells, which, under normal conditions, usually escape from immune surveillance by downregulating tumor antigen presentation [3] or by initiating immune tolerance [4], [5]. Presently, several antigens have been identified as potential immunotherapy candidates for prostate cancer vaccines. They include the prostate-specific antigen (PSA) [6], prostate stem cell antigen (PSCA) [7]–[10], prostate-specific membrane antigen (PSMA) [11], prostatic acid phosphatase (PAP) [2], mucin 1 (MUC1) [12], gonadotropin-releasing hormone (GnRH) [13], and NY-ESO-1 vaccine [14], among others. PSCA is a 123-amino acid glycosylphosphatidylinositol (GPI)-linked cell-surface protein belonging to the Ly-6 family [15]. PSCA is an attractive immunotherapeutic target based on its overexpression in a majority of prostate cancer cells, while its expression in other somatic tissues is highly limited [16]. Although the specific mechanism underlying the contribution of PSCA to tumor growth remains undefined, PSCA has been found to correlate positively with tumor malignancy, pathology grade and androgen-independence [16], [17]. It was suggested that PSCA might play a role in counteracting natural immune response [18]. Moreover, PSCA expression was upregulated in metastatic tissues [16], [19]. Currently, antibody directed to PSCA has been tested to inhibit prostate cancer tumor growth and suppress metastasis formation [20], while others have investigated chimeric antigen receptors (CAR)-based adoptive T cells therapy targeting PSCA for its potential in treating prostate cancer [21]. Experiments have also been conducted to test PSCA as a vaccine antigen, and it has been clearly shown in animal models that PSCA-targeted vaccines can slow down prostate cancer progression [22], [23].

Lentiviral vectors (LVs) are promising vectors for cancer immunotherapy [24], [25], and they are currently being evaluated in many clinical trials for a wide range of human diseases [26]. One desired trait of LVs is their ability to transduce both dividing and nondividing cells [27], including peripheral DCs [28], [29]. As a vaccine carrier, LVs can simultaneously deliver antigens to DCs [24] and activate DCs through toll-like receptors (TLRs) [30]–[36]. To further improve LVs, much effort has been directed toward targeting LVs to antigen-presenting cells (APCs) *in vivo* to achieve better specificity and safety [37]–[40]. We previously reported a DC-directed LV (DCLV), which can specifically target DCs expressing DC-specific intercellular adhesion molecule grabbing non-integrin (DC-SIGN) and deliver antigen genes to them. Direct *in vivo* vaccination using DCLV encoding chicken ovalbumin (OVA) elicited high frequency of OVA-specific CD8^+^ and CD4^+^ T cell responses [41]–[43].

In this report, we investigated DCLV-mediated cancer vaccines in a more clinically related setting and explored the potency of this vectored immunization to overcome the immune tolerance to the self-tumor antigen PSCA and to generate protective immunity against prostate cancer. We showed that DCLV encoding PSCA (DCLV-PSCA) could target DC-SIGN-expressing cell lines and bone marrow-derived DCs (BMDCs). Direct immunization at the base of the tail evoked strong PSCA-specific T cell responses in a mouse prostate cancer model. Furthermore, vaccination could significantly inhibit tumor growth upon challenge with TRAMP-C1 tumor cells in mice. When this vaccine was utilized in a therapeutic setting, it could suppress the growth of established TRAMP-C1 tumors. Our data showed that anti-prostate tumor immunity conferred by DCLV-PSCA depends on the presence of both CD8^+^ and CD4^+^ T cells. Finally, we demonstrated that immunization with DCLV-PSCA could efficiently inhibit the metastasis of B16-PSCA cells in lung tissue.

## Results

### Generation of DCLV-PSCA and its ability to target DC-SIGN-expressing cells in vitro

**Figure 1:**
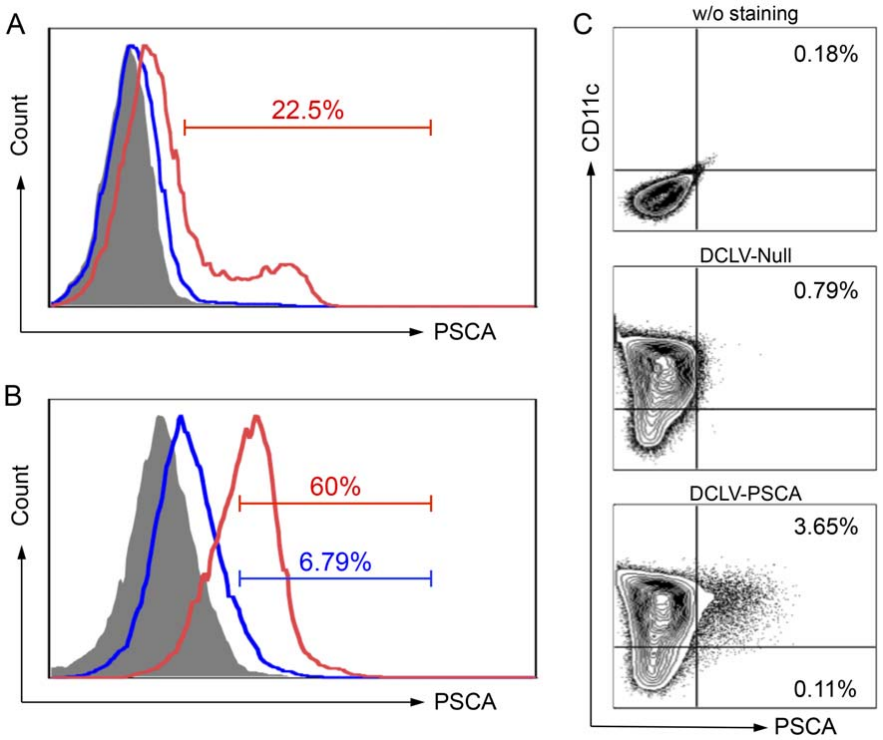
Targeted transduction and delivery of PSCA antigen gene into dendritic cells (DCs) by DCLV-PSCA. (A) 293T cells were transfected transiently with plasmids FUW-Null (mock control, blue line) or FUW-PSCA (red line). Two days later, cells were collected and stained for PSCA expression analyzed by flow cytometry. 293T cells stained with the isotype antibody were included as a control (grey shade area). (B) 293T cells were transfected transiently with plasmids FUW-PSCA, SVGmu, and other necessary lentiviral packaging plasmids to produce DCLV-PSCA vectors. Fresh virus supernatant was used to transduce 293T cells (blue line) or 293T.hDC-SIGN cells (red line) with MOI = 10. PSCA expression was analyzed by flow cytometry 3 days post-transduction. (C) Bone marrow-derived DCs were transduced with a mock vector DC-LV-Null or DC-LV-PSCA vector. Five days later, CD11c and PSCA expression were assessed by flow cytometric analysis. All experiments were repeated three times and the representative data is shown.

We constructed a lentiviral backbone encoding the full length of murine PSCA and tested the expression of PSCA in 293T cells. 293T cells were transiently transfected with FUW-Null or FUW-PSCA vector. Two days after transfection, the cells were collected for expression of PSCA by fluorescence-activated cell sorter (FACS) analysis. 293T cells transfected with the FUW-PSCA plasmid showed positive expression of PSCA (22.5%), while cells transfected with the FUW-Null plasmid had only background staining (Figure 1A).

We then utilized previously reported 293T.DC-SIGN cells [41] to investigate the ability of DCLV to express PSCA. As shown in Figure 1B, approximately 60% of the 293T.DC-SIGN cells displayed PSCA expression post-DCLV-PSCA transduction, whereas only 6.79% were PSCA-positive in the 293T cells. The specificity observed here is consistent with previous reports showing the ability of DCLV to preferentially transduce DC-SIGN-expressing cells [41], [44]. We further investigated whether DCLV-PSCA could target and mediate PSCA expression in bone marrow-derived DCs (BMDCs). The immature BMDCs were derived from the murine bone marrow culture and confirmed by flow cytometric analysis of cell surface marker CD11c (Figure 1C). When exposed to LVs, DCs were selectively modified by DCLV-PSCA to express PSCA (3.65% in the CD11c^+^ cells vs. 0.11% in the CD11c^−^ cells, Figure 1C). Our results indicated that DCLV-PSCA could target DC-SIGN-expressing cells and deliver the PSCA antigen to DCs *in vitro*.

### Induction of PSCA-specific CD8^+^ and CD4^+^ T cell immune responses in vivo

**Figure 2:**
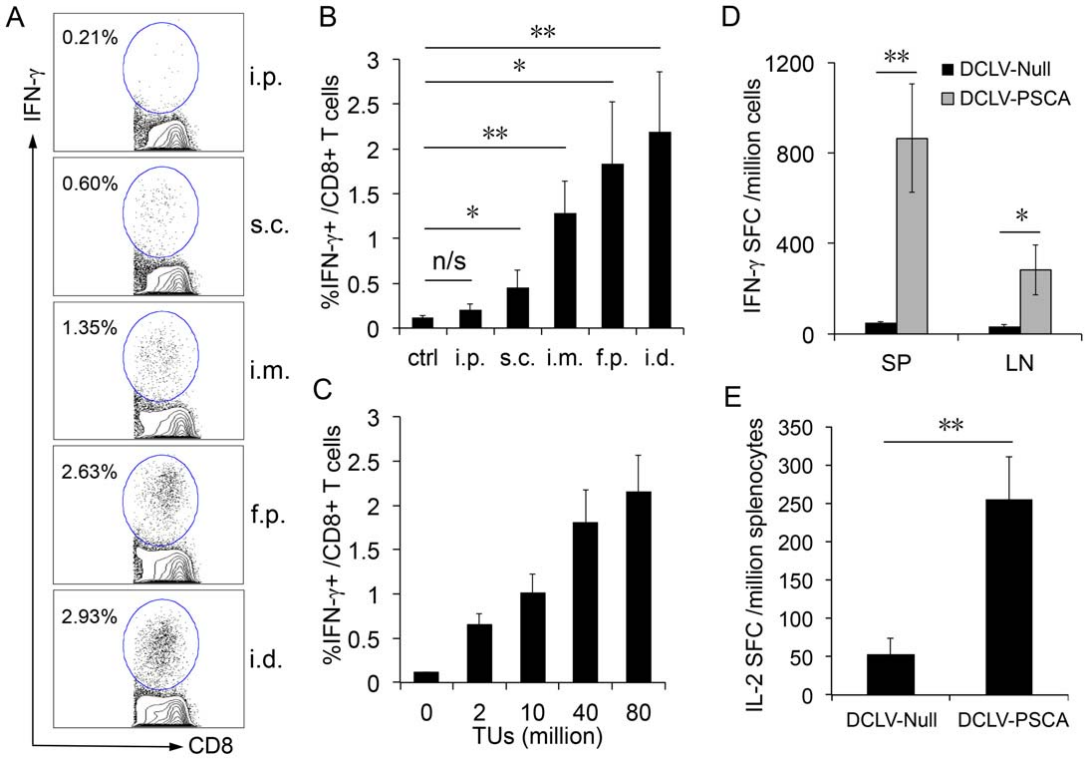
PSCA-specific T cell response after a single dose of *in vivo* immunization with DCLV-PSCA. (A) Male C57BL/6 mice were immunized with 6×10^7^ TU of DCLV-PSCA through different administration routes: intraperitoneal space (i.p.), subcutaneous area (s.c.), intramuscular area (i.m.), footpad (f.p.), or intradermal (the base of tail, i.d.). One immunization group was included as a negative control. Two weeks after immunization, splenocytes from mice were harvested and analyzed for the presence of PSCA-specific CD8^+^ T cells by restimulating splenocytes with a PSCA peptide (PSCA_83-91_), followed by intracellular staining for IFN-γ and surface staining for CD8. Percentage of IFN-γ-secreting CD8^+^ T cells is indicated. (B) Statistical comparison of immunization elicited by administration of DCLV-PSCA among different administration routes. (C) Male C57BL/6 mice were immunized with different doses of DCLV-PSCA vectors (0, 2, 10, 40 and 80 million TU) at the base of tail. Two weeks post-vaccination, PSCA-specific CD8^+^ T cells from the spleen were analyzed by restimulating with the peptide PSCA_83-91_, followed by intracellular staining for IFN-γ. (D) Production of PSCA-specific IFN-γ-secreting cells from both spleen (SP) and inguinal lymph node (LN) was evaluated by restimulation with the PSCA_83-91_ peptide, followed by ELISPOT analysis for IFN-γ. (E) Production of PSCA-specific IL-2 from splenocytes (with CD8^+^ T cells depleted) was measured by restimulation with 293T cell lysate transfected to express PSCA, followed by the ELISPOT analysis for IL-2. (**: *P*<0.01; *: *P*<0.05; One-way ANOVA followed by Bonferroni's multiple comparison test. Error bars represent SD.) All experiments were repeated three times and the representative data is shown.

To determine whether this recombinant DCLV-PSCA vector could efficiently deliver the antigen to DCs and mount antigen-specific T cell responses *in vivo*, we performed vaccination with DCLV-PSCA directly to male C57BL/6 mice. Because of the variation of DC distribution, immunization carried out through different administration routes may result in different numbers of DCs to be targeted, leading to different levels of antigen presentation. As such, comparison of the immunogenic response elicited through different routes is necessary to establish an optimal immunization protocol. Therefore, naïve male C57BL/6 mice were immunized with a single dose of DCLV-PSCA (6×10^7^ TU) at intradermal area (i.d., at the base of tail), footpad area (f.p.), intramuscular area (i.m.), subcutaneous area (s.c.), or intraperitoneal space (i.p.). A previously reported CD8^+^ epitope peptide for PSCA [23] was used to characterize PSCA-specific CD8^+^ T cell responses in the spleen via IFN-γ intracellular cytokine staining (ICCS). As depicted in Figure 2A and 2B, the i.d. and f.p. administration routes resulted in the strongest PSCA-specific CD8^+^ T cell response (∼2%) two weeks post-immunization. I.m. administration routes had reached a moderate response (∼1.2%) whereas the s.c. and i.p. injections resulted in a much lower response (<0.5%). This response trend is consistent with results from immunization with DCLV encoding HIV-1 Gag [45] or human gp100 [44]. Based on the i.d. administration route, which gave the highest CD8^+^ T cell response, different doses of DCLV-PSCA (2∼80×10^6^ TU) were administered. As shown in Figure 2C, the CD8^+^ T cell response was dose-dependent, increasing from 0.5% to 2%. Thus, an optimal immunization regimen of the i.d. injection of DCLV-PSCA with 80×10^6^ TU was employed for subsequent studies. To further assess the antigen-specific CD8^+^ T cell responses elicited by i.d. immunization, an ELISPOT experiment measuring IFN-γ secretion of T cells from both spleen and inguinal lymph node was conducted. Out of 1 million cells, approximately 800 and 300 cells responded to the CD8^+^ epitope peptide in the spleen and in the inguinal lymph node, respectively (Figure 2D).

Considering the important role of CD4^+^ in tumor immunotherapy, an IL-2 ELISPOT assay was employed to examine the CD4^+^ T cell response triggered by this immunization strategy. We detected approximately 250 CD4^+^ T cells per million splenocytes capable of secreting IL-2 in response to lysates from 293T cells transfected with the FUW-PSCA plasmid (Figure 2E). Our results demonstrated that DCLV-PSCA was efficacious as a vaccine carrier to stimulate both CD8^+^ and CD4^+^ T cell responses in mice.

### Generation of anti-prostate tumor immunity in both prophylactic and therapeutic models

**Figure 3:**
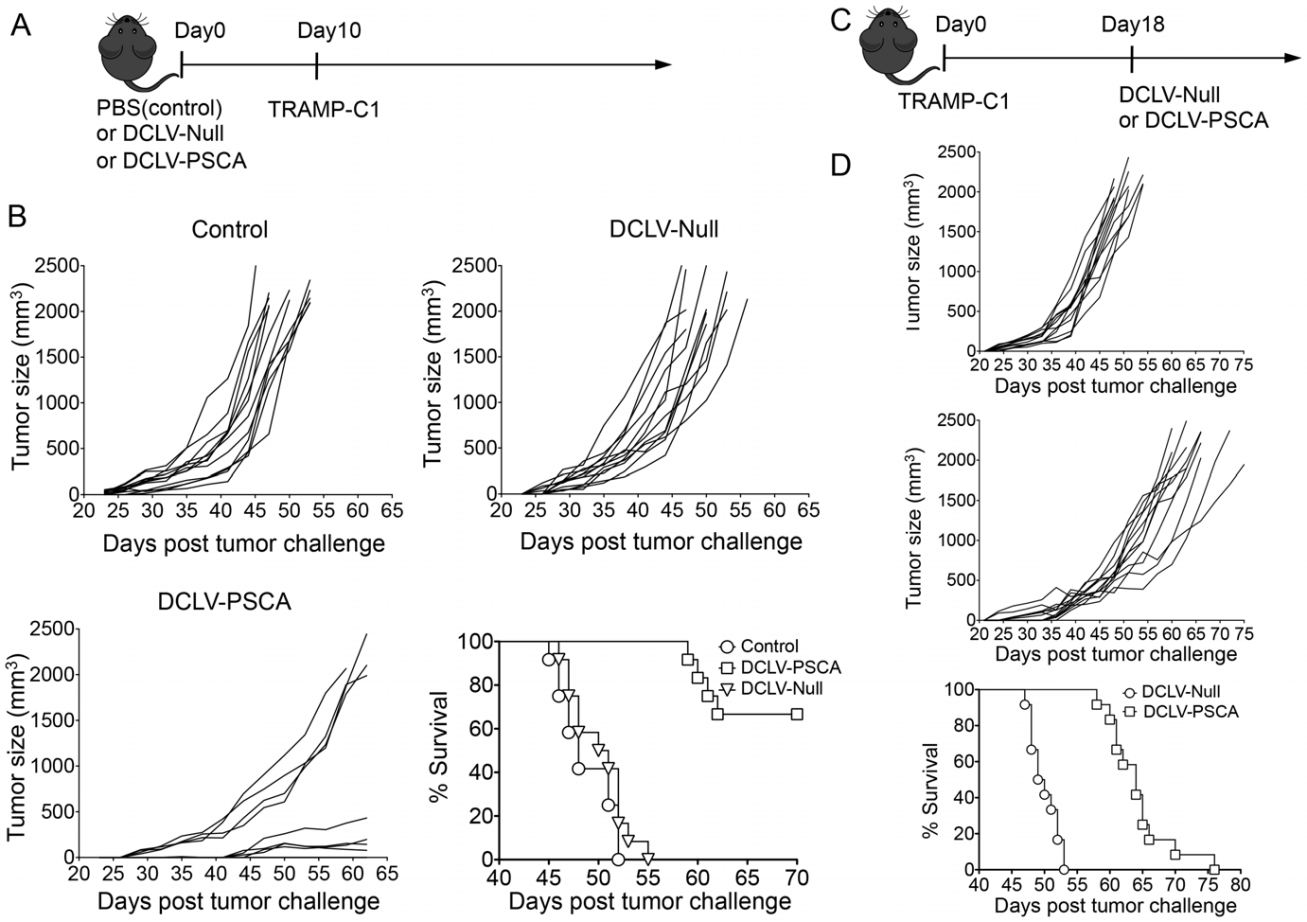
Prophylactic and therapeutic anti-TRAMP-C1 prostate cancer immunity elicited by *in vivo* immunization with DCLV-PSCA. (A, B) Male C57BL/6 mice were immunized with 8×10^7^ TU of DCLV-PSCA, mock vector DC-LV-Null, or PBS control at the base of tail. Ten days post-immunization, these mice were challenged subcutaneously with 5×10^5^ of TRAMP-C1 tumor cells. Tumor growth curves were monitored with a fine caliper, and tumor volume was calculated based on the largest perpendicular diameters (mm^3^), according to the formula *V = ab^2^π/6*, where *a* and *b* are the largest perpendicular diameters. Representative Kaplan Meyer survival curve for prophylactic tumor challenge (n = 12). (C, D) Male C57BL/6 mice were implanted with 5×10^5^ TRAMP-C1 tumor cells subcutaneously, and 18 days later, these tumor-bearing mice were treated with 8×10^7^ TU of DCLV-PSCA (n = 12) or DCLV-Null (n = 12) at the base of tail. Tumor volume was monitored and calculated as previously described. Representative Kaplan Meyer survival curve for therapeutic tumor challenge. (***: *P*<0.001; Log-rank (Mantel-Cox) test. Error bars represent SEM.) All experiments were repeated twice and the representative data is shown.

In light of the PSCA-specific CD8^+^ and CD4^+^ T cell response observed, it was necessary to evaluate the antitumor efficacy conferred by DCLV-PSCA immunization. A transplanted mouse tumor model with the transgenic adenocarcinoma mouse prostate cell line (TRAMP-C1) [46] was used for this evaluation. Male C57BL/6 mice were vaccinated with DCLV-PSCA, DCLV-Null, or left untreated. These mice were then challenged 10 days later by s.c. injection of 5×10^5^ TRAMP-C1 cells (Figure 3A). Tumor protection was observed in the DCLV-PSCA-vaccinated group with 8 out of 12 mice tumor-free for 44 days post-tumor challenge (Figure 3B, lower left). Moreover, the other 4 mice in that group exhibited a much slower rate of growth than that in the null vector group. Notably, vaccination with DCLV-Null failed to provide any measurable tumor suppression benefit as compared to the control group (Figure 3B, upper right to upper left). Overall, mice from the DCLV-PSCA group displayed a significantly better survival rate than that of mice from either the DCLV-Null or control group. All of the tumors from the DCLV-Null and control group exceeded the size limit within 55 days (the tumor size of 2000 mm^3^ was used as a surrogate endpoint of survival), whereas the DCLV-PSCA group survived more than 70 days (Figure 3B, lower right).

We further investigated whether DCLV-PSCA could be potent for inhibiting tumor growth in a therapeutic TRAMP-C1 model, in which a tumor had already been established (Figure 3C). Tumor-bearing mice therapeutically vaccinated with DCLV-PSCA showed significantly slower tumor growth (Figure 3D, upper and middle), and the average survival was extended from 49.5 days to 64 days following the DCLV-PSCA immunization (Figure 3D, lower).

### Dependence of vaccine-elicited antitumor immunity on infiltrated CD8^+^ and/or CD4^+^ T cells

**Figure 4:**
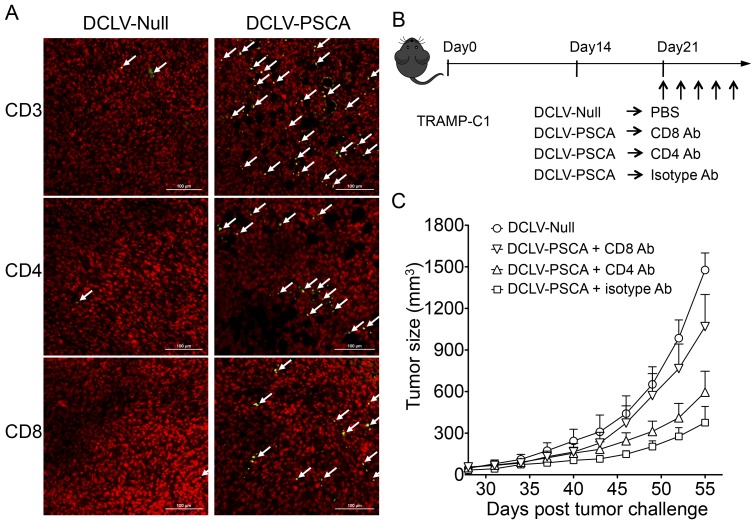
CD8^+^/CD4^+^ T cell-dependent immune protection against TRAMP-C1 tumors induced by DC-LV-PSCA immunization. (A) Infiltration of T cells into tumor tissues. TRAMP-C1 tumors from tumor-bearing mice were excised 3 weeks post-immunization, paraffin-embedded, and stained for immunofluorescence-conjugated CD3, CD4 and CD8 antibody (green color as indicated by white arrows) together with nuclear staining (red color). Representative images showing CD4^+^ and CD8^+^ T cells infiltrated to tumor tissues from DCLV-PSCA-immunized mice as compared to those of DCLV-Null-immunized mice. (B) Four groups of male C57BL/6 mice (n = 8 for each group) were transplanted with 5×10^5^ TRAMP-C1 cells subcutaneously at day 0. Fourteen days later, 3 groups were immunized with DCLV-PSCA, while the other group was immunized with mock vector DCLV-Null. Two groups of mice from the DCLV-PSCA-immunized groups were subjected to CD4^+^ or CD8^+^ T cell depletion by injecting CD4- or CD8-depletion antibody intraperitoneally. (C) Tumor volume for each group of mice was monitored. Error bars represent SEM. All experiments were repeated twice and the representative data is shown.

In an effort to further understand the roles of CD8^+^ and CD4^+^ T cells in antitumor immunity, tumor tissue samples from DCLV-Null- or DCLV-PSCA-immunized mice were collected, paraffin-embedded, and subjected to staining of nucleus and surface markers. As shown in Figure 4A, the immunization resulted in infiltration of more T cells (as identified by CD3 staining), including both CD4^+^ and CD8^+^ T cells in tumor tissues harvested from DCLV-PSCA-immunized mice, than that of DCLV-Null-treated mice. This indicates that both cytotoxic and helper T cells can infiltrate into the local tumor tissue in response to immunization. To determine the dependency of antitumor effect on these infiltrated T cells, an *in vivo* T cell depletion experiment was performed. Four groups of mice were inoculated with the TRAMP-C1 tumors, in which three groups were then immunized with DCLV-PSCA 14 days post-tumor challenge, while the remaining group was immunized with DCLV-Null. For the DCLV-PSCA-immunized groups, one group was treated with an antibody capable of depleting CD4+ T cells, and another group was treated with an antibody capable of depleting CD8^+^ T cells (Figure 4B). As shown earlier, DCLV-PSCA immunization could significantly slow down the overall tumor growth. In contrast, tumors in the groups with depletion of either CD8^+^ or CD4^+^ T cells developed a faster rate of tumor growth, although some tumor-protective effect remained. Notably, CD8^+^ T cell-depleted group had markedly larger tumors than that of the CD4^+^ T cell-depleted group (Figure 4C). Our data further indicate that T cells are responsible for the observed vaccine-induced antitumor immunity and that CD8^+^ T cells play the more indispensable role in controlling tumor growth.

### Protection against lung metastasis of B16-PSCA cells

**Figure 5:**
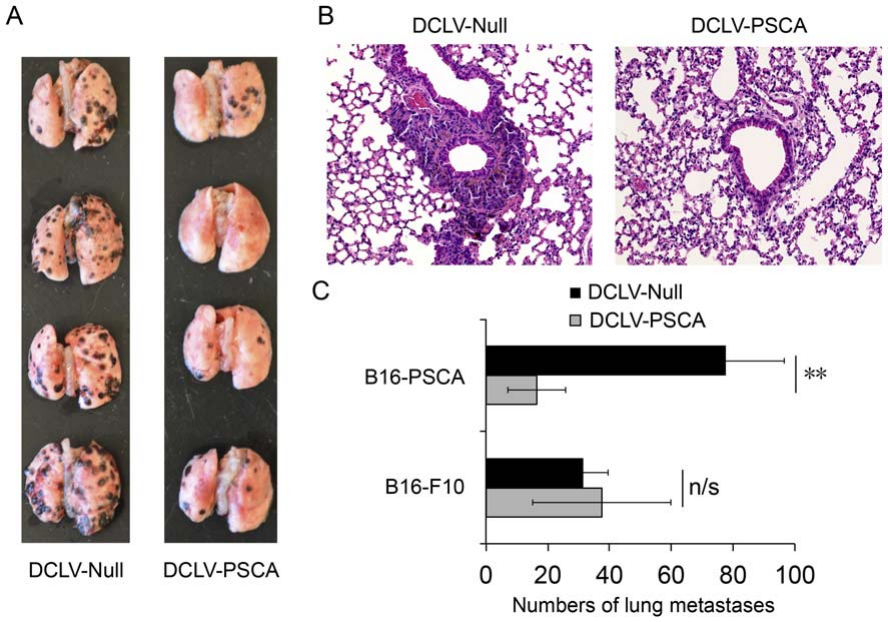
The ability of DCLV-PSCA immunization to suppress lung metastases. (A) Male C57BL/6 mice were immunized with DCLV-PSCA or DCLV-Null as a mock control. Ten days later, mice were challenged with 0.2 million B10-F10-PSCA cells by intravenous injection through tail vein. Two weeks later, mice were sacrificed, and macroscopic views of the lungs were shown. (B) Microscopic H&E staining (20×) of lung tissue samples from mice immunized with DCLV-PSCA or DCLV-Null. (C) Statistical quantification of melanoma lung metastases (number of black nodules on the lungs) of immunized mice; similar immunization, but with the original B16-F10 melanoma metastases included as a control. (**: *P*<0.01 and n/s: not statistically significant; One-way ANOVA followed by Bonferroni's multiple comparison test. Error bars represent SD, n = 4). All experiments were repeated twice and the representative data is shown.

Overexpression of PSCA was identified to be associated with prostate tumor metastasis in many studies, which makes it an ideal target for immunotherapy. To facilitate the study of the ability of DCLV-PSCA immunization to inhibit tumor metastasis formation, wild-type B16-F10 cells stably expressing PSCA was constructed (designated as B16-PSCA). Male C57BL/6 mice were first vaccinated with DCLV-PSCA or DCLV-Null as a negative control. Ten days later, syngeneic B16-PSCA tumor cells were injected intravenously to the animals. After another 14 days, animals were culled, and lung metastatic deposits were quantified macroscopically. Compared to DCLV-Null, DCLV-PSCA immunization markedly reduced the number of surface lung metastasis formation (>75%, Figure 5A and 5C). Histologic lung tissue samples from the two above groups were also examined microscopically for metastasis deposits, and a similar finding was observed (Figure 5B). In contrast, the protective immunity of DCLV-PSCA was only limited to the PSCA-expressing melanoma cells, as no significant difference was observed when B16-F10 tumor cells were transplanted (Figure 5C). These results confirmed PSCA-specific antitumor immunity conferred by DCLV-PSCA immunization and its capacity to suppress metastasis formation.

## Discussion

DC-based treatments have shown promising results for cancer immunotherapy [47], [48]. DC-directed LVs are efficient vaccine vectors. They are able to transduce and activate DCs *in vivo* and mediate durable transgene expression, which can be subsequently processed by DCs and presented to T cells as antigens [49]. Additionally, these vectors are engineered to be non-replicable, with minimal viral proteins being expressed, and, therefore, less anti-vector immunity was found [44]. Furthermore, because of DC-specific transduction, fewer safety and off-target concerns arise when DCs are applied as vaccine vehicles *in vivo* [41]. In our previous studies, we have demonstrated that DC-directed LVs (DCLVs) can elicit strong immune responses against OVA [50], HIV-gag [45], and hgp100 [44] antigens. In this study, we evaluated the DCLVs carrying PSCA, a true self-tumor antigen for prostate cancer, as a vaccine for syngeneic transplanted prostate tumor *in vivo*. To the best of our knowledge, this represents the first study to use DCLVs as a vaccine modality against a self-tumor antigen in animal models. We showed that DCLV-PSCA vaccination could overcome the tolerance to self-antigen PSCA and generate durable antigen-specific T cell responses *in vivo*. This immunization mounts an immune response that is capable of suppressing the establishment of TRAMP-C1 prostate tumors and slowing down tumor growth in a therapeutic model.

The envelope protein used to pseudotype LVs is an engineered form of Sindbis virus glycoprotein (SVGmu). The wild type of this glycoprotein has the binding affinity to both heparin sulfate and DC-SIGN; DC-SIGN is a surface protein that is predominantly expressed in macrophages and certain subsets of DCs [51]. We achieved targeting of DCs by disabling the heparin sulfate binding and retaining the DC-SIGN binding of the Sindbis virus glycoprotein. It has been demonstrated that the binding of SVGmu to DC-SIGN is dependent on the high mannose structure on DC-SIGN. Therefore, the viral glycoprotein can be further engineered to display a higher mannose structure to enhance transduction efficiency [52]. We first confirmed that DCLV-PSCA could be efficiently produced and selectively transduce DC-SIGN-expressing cells. The *in vitro* BMDC transduction assay substantiates the observation that DCLV-PSCA can direct the delivery of the PSCA antigen into DCs.

It has been previously shown that skin-derived DCs are the main target for LV-based vaccination [49]. However, the distribution and accessibility of DCs in different parts of the body vary, so immunization through different routes might trigger different levels of immune responses. We previously reported that the f.p. route had a relatively higher response over other administration routes for some antigen deliveries [44], [45]. In this study, we directly compared immune responses elicited through various vaccination routes (i.d., f.p., i.m., s.c. and i.p.). Interestingly, we found that i.d. and f.p injections generated much higher responses than did the i.m. and s.c route, whereas i.p. administration resulted in the lowest response. Immunization generated through the i.d. route displayed a slightly higher response than the f.p. route. To account for this result, it is speculated that DCLV-PSCA has a better chance of encountering DCs when administered through either the i.d. or f.p. route.

A single dose of DCLV-PSCA was able to protect these mice from prostate tumor challenge and improved their survival rate. This result is consistent with a previous study using a prime/boost strategy to generate PSCA-specific immune response in a prophylactic model [22], [23], although DCLV-PSCA elicited a higher magnitude CD8^+^ T cell response. In a TRAMP-C1 therapeutic model, our vectored vaccine markedly slowed down tumor growth and extended mouse survival, whereas the previous prime/boost vaccine method barely generated satisfactory tumor protection [22]. This result can be explained by the time interval between tumor inoculation and tumor palpability, a period of around 20∼25 days. Also, it takes a significantly longer period of time to implement the prime/boost immunization, which likely results in missing the most opportune time to slow down cancer progression. Thus, one potential advantage of DCLVs is their ability to overcome immune tolerance and establish an effective antitumor immune response within 2 weeks.

Both CD8^+^ and CD4^+^ T cells infiltrated into local tumor tissues following DCLV-PSCA immunization. Although CD8^+^ and CD4^+^ T cells are both required for tumor protection, the antibody depletion experiment indicates that CD8^+^ T cells play a more important role in controlling tumor growth. It has been well established that cytotoxic CD8^+^ T cells can directly kill tumor cells [53]–[55]. As for the CD4^+^ T cells, several possible reasons explain their requirement for tumor protection. First, CD8^+^ T cells are dependent on CD4^+^ T cells [56], [57] to elicit robust immune responses. Previously, we observed a CD4-dependent CD8^+^ T cell response that was elicited by DCLVs [58]. Second, at least part of the antitumor effect is mediated by the Th1 response, which relies on CD4^+^ T cells [59].

Currently, no reliable treatment exists to cure advanced metastatic prostate cancer. PSCA is highly expressed in metastatic tissue for prostate cancer and is therefore a good target for cancer immunotherapy. We have shown in this study that DCLV-PSCA can generate immunity able to suppress lung metastasis in the B16-PSCA model. Interestingly, when the same number of B16-F10 or B16-PSCA cells was injected intravenously, B16-PSCA cells were able to generate more lung metastasis formation than that of B16-F10 cells (Figure 5C). Presently, the relationship between tumor metastasis and PSCA expression has not been thoroughly investigated, although some studies suggest that PSCA may play a role in limiting tumor migration and metastasis [60]. Nevertheless, more studies are needed to further understand how PSCA expression contributes to prostate cancer metastasis, and B16-PSCA might be a suitable model for such studies.

Taken together, we have reported a novel DCLV vector system that can deliver self-tumor antigen PSCA to antigen-presenting cells and mount vaccine-specific immune responses. This DCLV-PSCA can overcome immune tolerance to PSCA, generate T cell immunity that can protect mice in TRAMP-C1 prostate tumor models, and significantly inhibit B16-PSCA lung metastasis formation. These results offer evidence to support the use of DCLVs to deliver prostate cancer vaccines.

## Materials and Methods

### Mice and cell lines

Male C57BL/6 mice (6–8 weeks old) were purchased from the Charles River Laboratories (Wilmington, MA, USA). All mice were maintained in the animal facilities at the University of Southern California (USC) under controlled temperature and a 12 h light/dark cycle, with free access to water and standard laboratory chow. Animal procedures were performed in accordance with the guidelines set by the National Institutes of Health (NIH Publication No. 85-23, revised 1996) and the animal protocol was approved by the Institutional Animal Care and Use Committee of the USC (2010-11450). The tumor size of 2000 mm^3^ was used as a surrogate endpoint of survival, and mice will be euthanized by CO2 inhalation from a tank source and a follow-up cervical dislocation. TRAMP-C1 cells were obtained from ATCC (Manassas, VA, USA) and cultured in DMEM high glucose (Cellgro, Manassas, VA, USA) with L-glutamine supplemented with 5% FBS, 5% Nu Serum IV (BD Biosciences, San Jose, CA, USA), bovine pancreas insulin 5 µg/ml (Sigma, St. Louis, MO, USA) and 10 nM dehydroisoandrosterone (ChromaDex, Irvine, CA, USA). B16-F10 cells were purchased from ATCC (Manassas, VA, USA) and cultured in DMEM high glucose (Cellgro, Manassas, VA, USA) with L-glutamine supplemented with 10% FBS. B16-F10 cells stably expressing PSCA were generated by transducing B16-F10 cells with lentivirus (FUW-PSCA) pseudotyped with vesicular stomatitis virus glycoprotein (VSVG), and clonal cells were selected.

### Construction and production of lentiviral vectors

The lentiviral backbone plasmid FUW-PSCA was constructed by insertion of the cDNA of murine PSCA downstream of the ubiquitin promoter in FUW. FUW is a HIV-1-derived lentiviral plasmid composed of an internal human ubiquitin-C promoter to drive transgene expression and woodchuck responsive element to improve stability of the RNA transcript [61]. We employed a previously reported procedure of transient transfection of 293T cells to produce the DCLV-PSCA vector [41]. Briefly, 293T cells cultured in a 15-cm tissue culture plate (BD Biosciences, San Jose, CA, USA) were transfected via a standard calcium phosphate precipitation method with the following plasmids: the lentiviral backbone plasmid FUW-PSCA (37.5 µg, Figure 1A), the plasmid encoding the mutant Sindbis virus glycoprotein (SVGmu, 18.75 µg, Figure 1B), and the packaging plasmids (pMDLg/pRRE and pRSV-Rev, 18.75 µg each). The viral supernatants were harvested twice at 48 and 72 hrs post-transfection, pooled, and filtered through a 0.45-mm filter (Corning, Lowell, MA, USA). The concentrated viral pellets were obtained after ultracentrifugation of the viral supernatants at 50,000 ×g for 90 min and were then resuspended in an appropriate volume of HBSS for *in vivo* administration.

### BMDC generation and staining

Bone marrow-derived DCs (BMDCs) were generated according to a previously described procedure [41]. Briefly, bone marrow from the femurs and tibias of male C57BL/6 mice was grown in RPMI 1640 with 10% heat-inactivated FBS, 2 mM L-glutamine, 100 U/ml penicillin, 100 µg/ml streptomycin, 0.05 mM 2-ME, and 20 ng/ml GM-CSF (J558L supernatant) after the red blood cells were lysed. Cultures were initiated by placing 10^7^ bone marrow cells in 10 ml of medium onto 100-mm petri dishes (Falcon 1029 plates; BD Labware, Franklin Lakes, NJ). On day 3, another 10 ml of J558L-conditioned medium were added. On day 6, suspension cells were collected. BMDCs were seeded at a density of 0.5 million/ml in 24-well plates (BD Labware) and spin-transduced twice with either DC-directed LV without antigen insertion (DCLV-Null) or DCLV-PSCA at 2500 rpm and 25°C for 90 min. Five days later, BMDCs were collected and incubated with anti-mouse CD16/CD32 Fc blocking antibody and then stained with rabbit anti-mouse PSCA (clone M-70, Santa Cruz Biotechnology, Santa Cruz, CA, USA) at 4°C for 20 min. After a washing step, BMDCs were further incubated with donkey anti-rabbit IgG-PE (Abcam, San Francisco, CA, USA) and anti-CD11c-PE-Cy5 (BioLegend, San Diego, CA, USA) at 4°C for 10 min, followed by washing and analysis by BD LSRII flow cytometer (BD Biosciences). Acquired data were analyzed using FlowJo software (Tree Star, Ashland, OR).

### In vivo depletion of CD4^+^or CD8^+^ T cells

Four groups of mice were implanted with 5×10^5^ TRAMP-C1 cells subcutaneously at day 0. Fourteen days later, three groups of mice were injected with 8×10^7^ transduction units (TU) of replication-defective DC-LV-PSCA at the base of tail. At day 21, 24, 27, 30 and 33, each group of immunized tumor-bearing mice was intraperitoneally injected with one of the following antibodies: 200 µg CD4 antibody (clone GK1.5, BioXCell, West Lebanon, NH), 200 µg CD8 antibody (clone 53.6.72, BioXCell), or 200 µg isotype antibody (BioXCell). Tumor growth was monitored.

### IFN-γ intracellular cytokine staining (ICCS)

Splenocytes from immunized or control mice were pooled and incubated with the PSCA_83-91_ peptide (NITCCYSDL) (GenScript, Piscataway, NJ, USA) at final concentration of 50 µg/ml for 2 h at 37°C in a 96-well round-bottom plate in RPMI medium supplemented with 10% FBS (Sigma), 10 U/ml penicillin, 100 µg/ml streptomycin, and 2 mM glutamine. Brefeldin A (BFA, Sigma, St. Louis, MO) was added (10 µg/ml) to wells to inhibit cytokine exporting for another 6 h. Surface staining was performed by incubating restimulated cells with anti-mouse CD16/CD32 Fc blocking antibody, followed by anti-mouse CD8 and anti-mouse CD4 antibodies. Cells were then permeabilized in 100 µl Cytofix/Cytoperm solution (BD Biosciences) at 4°C for 10 min, washed with Perm/Wash buffer (BD Biosciences), followed by intracellular staining with PE-conjugated anti-mouse IFN-γ at 4°C for 15 min. Flow cytometry analysis was carried out using the FACSort instrument from BD Biosciences.

### Enzyme-linked immunosorbent spot (ELISPOT) assay

To measure PSCA-specific CD8^+^ T cell responses, ELISPOT assays were performed to detect IFN-γ using a kit from Millipore (Billerica, MA) according to the manufacturer's instruction. Briefly, anti-mouse IFN-γ antibody (10 µg/mL in PBS) was used as the capture antibody and plated with 100 µl/well on 96-well MultiScreen-IP plates overnight at 4°C. The plate was decanted and blocked with the RPMI medium containing 10% FBS at 37°C for 2 h. Splenocytes from vaccinated mice were plated at 2×10^5^ cells/well in 100 µl complete medium in the presence of the CD8 epitope PSCA_83-91_ peptide (50 µg/ml). After 18 h incubation at 37°C, cells were lysed, and plates were detected by 1 µg/ml biotinylated anti-IFN-γ antibody (BD Biosciences) for 2 h at room temperature. Plates were further washed and incubated with the 1,000-fold-diluted streptavidin-alkaline phosphate conjugate for 45 min at room temperature. After a final extensive wash, spots were identified by adding BCIP/NBTplus substrate (Millipore), and the number of IFN-γ-producing cells was quantified by an ELISPOT reader. An IL-2 ELISPOT assay was also performed to examine PSCA-specific CD4^+^ T cell responses. The entire procedure is similar to the IFN-γ ELISPOT assay, except that IL-2 capture and detection antibodies were used instead; splenocytes with CD8^+^ T cells depleted using CD8 MicroBead Kit (Miltenyi Biotec, Auburn, CA, USA) were co-cultured for 40 h with lysates from 293T cells transfected with the FUW-PSCA plasmid.

### Histological analysis

TRAMP-C1 tumor-bearing mice were injected with DC-LV-PSCA (8×10^7^ TU at the base of tail) or untreated as a control. Twenty days later, tumors were excised, paraffin embedded and sectioned (5 µm thickness). The following antibodies were employed to detect tumor-infiltrating lymphocytes: anti-CD3-Alexa488 (clone 17A2 from BD Biosciences, San Jose, CA, USA), anti-CD4-Alexa488 (clone RM4-5 from BD Biosciences), anti-CD8-FITC (clone 53-6.7 from BD Biosciences). TO-PRO-3 (Invitrogen, Carlsbad, CA, USA) was used for nucleus staining. For the B16-PSCA metastasis experiment, lungs from the mice were excised, paraffin embedded, sectioned (5 µm thickness), and H&E stained. Samples were then analyzed microscopically with a 20× objective.

### Statistics

All the statistics were calculated by either Origin Pro 7.0 or GraphPad Prism 5 software. Error Bars in all the figures represent SD, except for the tumor growth curves in the prophylactic and therapeutic tumor challenge models, in which SEM was used. One-way ANOVA followed by Bonferroni's multiple comparison test was used to determine the significance of difference, while animal survival curves were analyzed by log-rank (Mantel-Cox) test, and the value of *P*<0.05 was considered to be statistically significant.
